# The Lupus Attack: A Case Report and Literature Review of Myocardial Infarction and Antiphospholipid Syndrome

**DOI:** 10.7759/cureus.24067

**Published:** 2022-04-12

**Authors:** Abhinav Karan, Adefemi Adeyemo, Michael Omar, Kerolos Fahmi, Srinivasan Sattiraju

**Affiliations:** 1 Internal Medicine, University of Florida College of Medicine, Jacksonville, USA; 2 Cardiology, University of Florida College of Medicine, Jacksonville, USA; 3 Interventional Cardiology, University of Florida College of Medicine, Jacksonville, USA

**Keywords:** cardiac chest pain, heart failure with reduced ejection fraction, cardiolipin antibody, acute coronary thrombosis, antiphospholipid antibody (apla), dual-antiplatelet therapy (dapt), primary pci, st-elevation myocardial infarction (stemi), systemic lupus erythematosis, lupus

## Abstract

Acute myocardial infarction in a young patient is a nebulous entity, but in the absence of traditional cardiovascular risk factors, particular attention must be paid to thrombotic disorders and hypercoagulable states. A 28-year-old male presented with worsening substernal chest pain for 36 hours. He was recently diagnosed with systemic lupus erythematosus (SLE) with active class II lupus nephritis. With an initial electrocardiogram revealing ischemic changes, and an elevated troponin I, a concern was raised for myocardial infarction. Transthoracic echocardiography revealed a severely reduced ejection fraction of 25%, and a subsequent emergent left heart catheterization revealed a complete, massive thrombotic occlusion of the proximal left anterior descending artery, requiring aspiration thrombectomy. After extensive workup for hypercoagulable states, he was found to have elevated anticardiolipin IgG and IgM antibodies on two occasions, twelve weeks apart. The patient was managed with triple anticoagulation with aspirin, clopidogrel, and warfarin for one month, followed by dual anticoagulation with clopidogrel and warfarin with a targetted international normalized ratio (INR) of 2.0 - 3.0. The management of acute coronary syndrome caused by antiphospholipid syndrome (APS) is highly individualized and driven by clinician gestalt owing to the lack of a standardized consensus. While systemic thrombolysis, primary percutaneous coronary intervention (PCI), and coronary artery bypass grafting all have their utility, only a very small handful of case reports exist on the benefits of each. This particular case serves to showcase an instance where a patient was successfully managed with PCI with dual antiplatelet therapy. Further prospective randomized controlled trials are necessary to determine the optimal management of this rarely encountered patient population.

## Introduction

Antiphospholipid syndrome (APS) is characterized clinically by a vascular thrombotic or maternofetal morbidity event, in the presence of an elevated antiphospholipid antibody on two separate laboratory tests done 12 weeks apart. It is an uncommonly encountered disease process, but an important etiologic consideration in a young patient with myocardial infarction in the absence of traditional cardiovascular risk factors. Here we describe the management of a young male with systemic lupus erythematosus (SLE) who presented with acute myocardial infarction, alongside a review of the corresponding literature. 

## Case presentation

**Figure 1:**
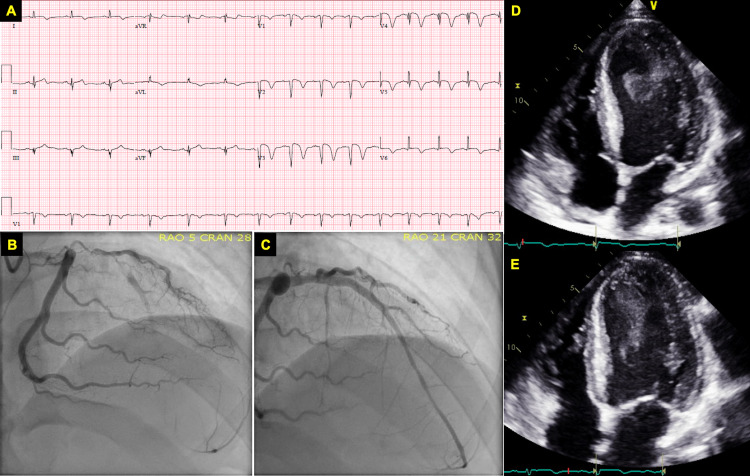
A) 12-lead electrocardiogram showing deep Q waves and 1 mm ST segment elevations anteroseptally with anteroseptal and lateral T wave inversions. B) Apical 4-chamber view on transthoracic echocardiogram showing significant spontaneous echo contrast in the left ventricle at end diastole and (C) end systole. D) Coronary angiogram showing thrombotic occlusion of the proximal left anterior descending artery followed by (E) reperfusion of the artery after aspiration thrombectomy.

A 28-year-old African-American male presented with worsening substernal chest pain for 36 hours. His past medical history was significant for a recent diagnosis of SLE with active class II lupus nephritis diagnosed through a renal biopsy, three months prior to presentation. He had remained compliant with his prescribed hydroxychloroquine and prednisone therapy since diagnosis. He was hemodynamically stable and the physical examination was unremarkable. The initial electrocardiogram revealed deep Q waves and 1 mm ST-segment elevations anteroseptally, with T wave inversions in the anteroseptal and lateral leads. Conventional troponin I was elevated. An immediate transthoracic echocardiogram showed a severely reduced left ventricular (LV) ejection fraction of 20%-25%. There was apical akinesis and significant swirling of spontaneous echo contrast suggestive of stasis and a very high risk for LV thrombus formation. Coronary angiography revealed a complete, massive thrombotic occlusion of the proximal left anterior descending artery, with a thrombolysis in myocardial infarction (TIMI) score of 0, consistent with no distal blood flow. The remaining vessels were angiographically normal. Attempts at balloon dilatation with tirofiban and cangrelor therapy failed at restoring coronary flow. Ultimately, aspiration thrombectomy was successful in reducing the thrombotic burden, and a drug-eluting stent was deployed with an excellent end result, with TIMI-3 reperfusion, and resolution of the patient's angina (Figure 1).

An initial workup consisted of a normal lipid panel, with low-density lipoprotein cholesterol (LDL-C) of 46 mg/dl (reference range: <70mg/dl), HbA1c of 5.1%, negative urine toxicology, negative HIV and syphilis panel, mildly elevated erythrocyte sedimentation rate of 37 mm/hr (reference range: 0-15 mm/hr), and no evidence of underlying sickle cell disease. C3 and C4 complement levels were mildly reduced. The patient himself endorsed no tobacco use, substance use, or over-the-counter or herbal supplements. At this stage, atherosclerotic plaque rupture was lower on the list of differential diagnoses due to the absence of traditional cardiac risk factors, which also included hypercoagulable states, coronary vasculitis, spontaneous coronary artery dissection, or even embolization from Libman-Sacks endocarditis.

He underwent an extensive workup comprising of a renal function panel, systemic autoimmune screen, and relevant inpatient thrombophilia testing. He was found to have positive antinuclear antibodies (ANA), anti-double-stranded DNA (anti-dsDNA), and anti-Smith antibodies consistent with the patient's known history of SLE, but his remaining systemic autoimmune screen was negative, including a negative p-antineutrophil cytoplasm antibodies (ANCA), c-ANCA, anti-Scl 70, rheumatoid factor, anti‐cyclic citrullinated peptide (anti‐CCP), and Sjogren's antibodies. He was screened for APS, revealing negative anti-B2-glycoprotein antibodies, but an elevated anticardiolipin IgG antibody titer at 60 GPL U/ml, consistent with moderate titers (40-79 GPL U/ml). Lupus anticoagulant (LAA) was not tested given the recent anticoagulation and negative COVID-19 testing. Given the high clinical suspicion of secondary APS, the patient was initially managed with triple antithrombotic therapy for one month using aspirin, clopidogrel, and warfarin. This was followed by lifelong dual therapy using clopidogrel and warfarin, targeting an international normalized ratio (INR) of 2.0-3.0. Laboratory classification for APS was later confirmed with a second set of titers, Twelve weeks from initial testing, laboratory classification for APS was confirmed with a second set of anticardiolipin IgG antibody titer of 54 GPL U/ml, consistent with moderate titers. Over the ensuing 12 months, there were no further thrombotic events.

## Discussion

APS is associated with multiple cardiac manifestations including valvulopathies, myocardial dysfunction, pulmonary hypertension, early bypass graft failure, intracardiac thromboses, and microvascular and macrovascular coronary artery disease. In the largest cohort of 1000 patients with APS, the prevalence of acute myocardial infarction was noted at 5.5%, with 2.8% of patients presenting with acute myocardial infarction as the first clinical manifestation of APS [1]. Secondary APS has been shown to carry a higher annual risk of thrombosis compared to primary APS but with no particular distinction in coronary events [1,2].

The commonly identified antiphospholipid antibodies are LAA, anticardiolipin IgG or IgM, and anti-B2-glycoprotein I IgG (aB2GPI). These pathogenic antibodies deposit on valvular or vascular endothelium, activating an inflammatory cascade that leads to valvular dysfunction, microvascular injury, and premature accelerated atherosclerosis [3,4]. Yet, reports of acute myocardial infarction associated with APS are all characteristically acute thrombotic events [3]. Moreover, even intracardiac thrombus formation has been linked particularly to anticardiolipin IgG, compared to other antibody profiles, as in our patient [4].

There are data to suggest a correlation between the risk of primary and recurrent coronary events to particular antibody profiles such as LAA positivity, multiple or persistent antibody positivity, or high anticardiolipin titers [5]. However, this remains a highly controversial area, as exposed in our case, of a high thrombus burden with moderate titers of a single antibody [2]. The literature is heterogeneous, with a scarcity of randomized controlled trials, but with unanimous agreement that all cases of APS should be treated with the same standard, regardless of the antibody titer. Aggressive traditional cardiovascular risk factor modification is unanimously agreed upon given the most recent recommendations by the European League Against Rheumatism (EULAR) endorsing the consideration of low dose aspirin for primary thromboprophylaxis in SLE patients with antiphospholipid antibodies [6]. 

The management of myocardial infarction in APS is based on conventional standards and clinician-specific gestalt, with nuances from observational studies, as there are no particular guidelines addressing the issue. Historically, systemic thrombolysis had demonstrated successful results and may still have a role in situations such as multiple presenting thrombi or catastrophic antiphospholipid syndrome (CAPS) [2,7]. There are very limited observational studies and case reports suggesting poorer outcomes of primary percutaneous coronary intervention (PCI) and coronary artery bypass grafting in patients with APS due to recurrent stent thrombosis and graft failure [2,8]. However, primary PCI and dual antiplatelet therapy are still the standard of care for APS patients with ST-elevation MI [2].

Vitamin K antagonists (VKA) have been successful in preventing recurrent arterial thromboses in APS as opposed to direct oral anticoagulants and are supported by the EULAR as monotherapy for secondary thromboprophylaxis with a goal INR of 2.0-3.0 [9]. In select cases such as therapeutic failure, a higher INR target of 3.0-4.0, the addition of aspirin, or switching to low molecular weight heparin may be considered [9]. In our case, early and aggressive triple antithrombotic therapy was initiated with warfarin in addition to dual antiplatelet therapy, given our patient’s high thrombotic risk and relatively low bleeding risk. This was extrapolated from extensive data on PCI in patients with atrial fibrillation [10]. After one month, the patient was de-escalated to a P2Y12 inhibitor plus warfarin for a subsequent year with success in preventing further thrombotic events.

## Conclusions

Acute myocardial infarction in a young patient must raise concern for APS as there are additional and still poorly understood caveats to management. High-quality evidence on treatment within this niche is lagging behind the plethora of novel coronary intervention techniques, stent designs, antithrombotic agents, and combination strategies. In summary, while PCI with dual antiplatelet therapy appears to be the standard of therapy, further randomized controlled trials are necessary to determine other variables, including the duration of therapy, benefits of triple anticoagulation, and the overall effectiveness of other modalities of therapy. Acute myocardial infarction in young patients remains an entity that requires careful consideration, both for its diagnosis and for its individualized management. 
